# Tuberculous Hypertrophic Pachymeningitis

**DOI:** 10.7759/cureus.17570

**Published:** 2021-08-30

**Authors:** Nikhil L Cordeiro, Sushilkumar S Gupta, Anubhav Kanwar, Carolyn Bendor-Grynbaum, Jyoti B Sharma

**Affiliations:** 1 Cardiology, Maimonides Medical Center, Brooklyn, USA; 2 Critical Care, Maimonides Medical Center, Brooklyn, USA; 3 Infectious Disease, Tri-State Memorial Hospital, Clarkston, USA; 4 Internal Medicine, Maimonides Medical Center, Brooklyn, USA; 5 Neurology, Fortis Hospital, Noida, IND

**Keywords:** hypertrophic, cranial, pachymeningitis, dura, tuberculosis, dural, thickening, enhancement, brain awareness

## Abstract

Tuberculous pachymeningitis is a rare disease that should be suspected in patients with chronic headaches, focal neurological signs, and magnetic resonance imaging (MRI) findings of dural thickening. We report the case of a 62-year-old male who presented with chronic headaches for over a year, progressive right-sided vision and hearing loss for six months, and progressive dysphagia for a month. On investigation, MRI showed dural thickening, cerebrospinal fluid showed lymphocytic pleocytosis with high protein and normal glucose levels, and biopsy of the dural matter showed necrotizing granulomas with Langhans giant cells. The patient responded well to antitubercular treatment and steroids. Follow-up MRI 24 months later showed almost complete resolution of meningeal enhancement. Though tuberculosis is an uncommon cause of pachymeningitis, it should be considered, as it responds well to treatment.

## Introduction

Meningeal hypertrophic thickening can occur due to various pathological processes such as infection, malignancy, rheumatic disease, hemodialysis, mucopolysaccharidosis, and intrathecal drug administration [1]. Meningeal involvement may be diffuse or focal. Some diseases involve only the dura mater and others predominantly involve the leptomeninges [1]. Pachymeningitis is a rare disease that is characterized by inflammation and fibrosis leading to localized or diffuse thickening of the dura mater. It can be visualized on magnetic resonance imaging (MRI) as contrast enhancement thickening of the dura mater [2]. Pachymeningitis can be cranial or spinal. Cranial pachymeningitis typically presents with symptoms of chronic headaches, facial pain, cranial nerve defects, and cerebellar ataxia. Spinal pachymeningitis presents with symptoms of nerve root compression. A biopsy of the dura mater helps confirm the diagnosis. Treatment of pachymeningitis is usually with corticosteroids and treatment of the underlying etiology [3,4].

## Case presentation

**Figure 1:**
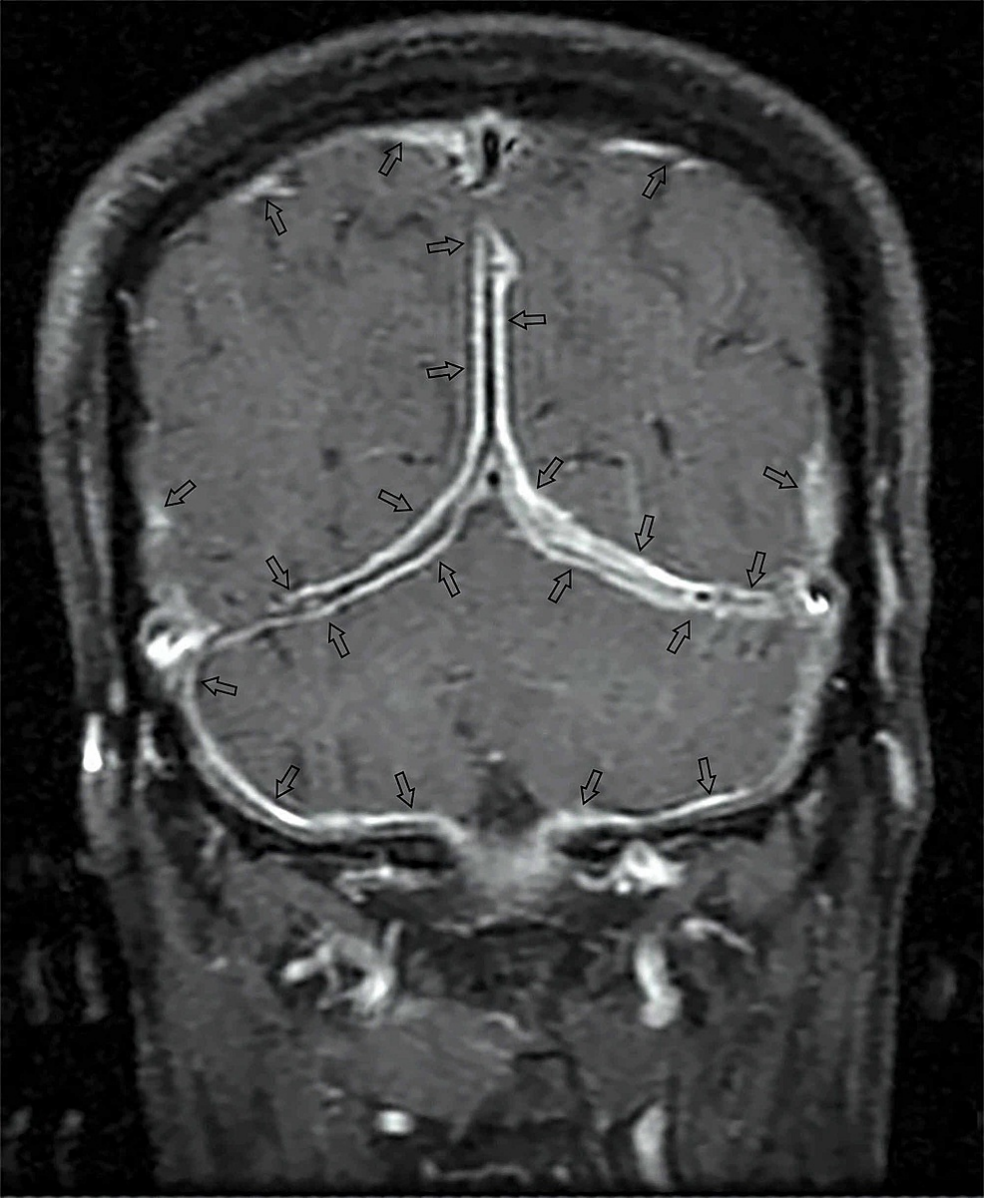
Pretreatment: Post-gadolinium coronal T1-weighted MRI showing diffuse posterior dural enhancement. Arrows show dural enhancement. MRI: magnetic resonance imaging

A 62-year-old male with a history of pulmonary tuberculosis presented with headaches for one year, progressive right-sided visual and hearing loss for six months, and progressive dysphagia for one month. Examination showed multiple cranial neuropathies: II, VI, and VII cranial nerves on the right, and bilateral VIII, IX, and X cranial nerves. Fundoscopy revealed a pale optic disc with no papilledema and optic atrophy in the right eye. MRI brain on presentation showed diffuse posterior pachymeningeal enhancement suggestive of dural thickening (Figure 1).

**Figure 2:**
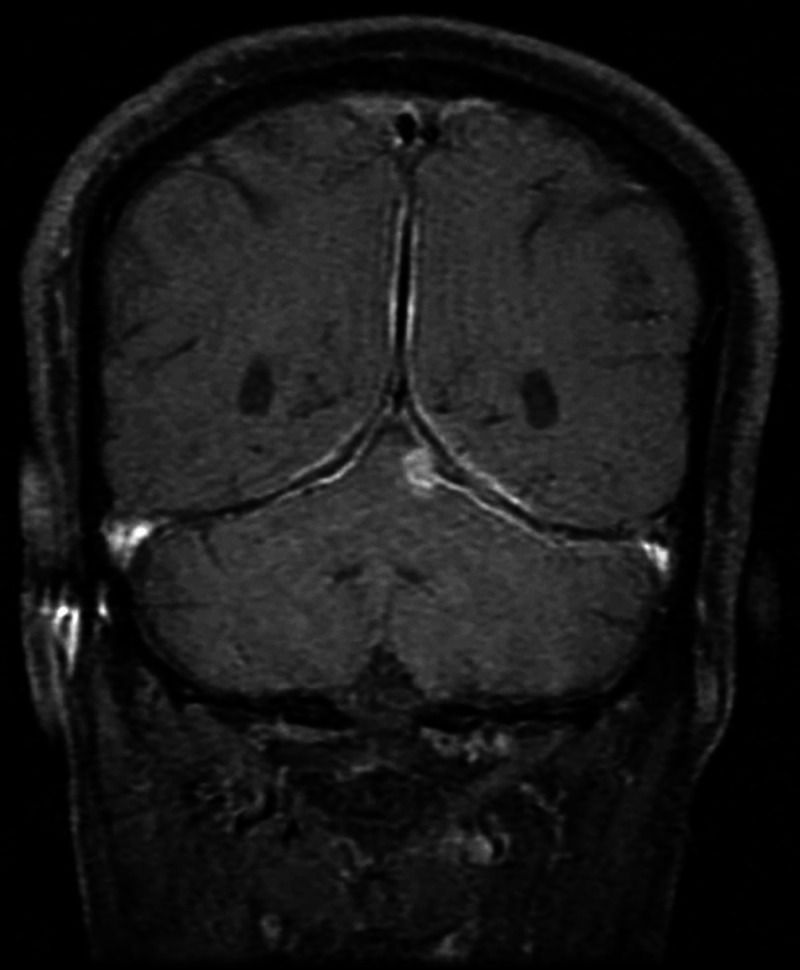
Posttreatment at 24 months: Post-gadolinium coronal T1-weighted MRI showing almost complete resolution of dural enhancement. MRI: magnetic resonance imaging

Cerebrospinal fluid (CSF) analysis showed lymphocytic pleocytosis, with high normal protein and normal glucose levels. Workup for vasculitis, which included perinuclear antineutrophil cytoplasmic antibodies, cytoplasmic antineutrophil cytoplasmic antibodies, antinuclear antibodies, Venereal Disease Research Laboratory test, was negative and angiotensin-converting enzyme levels were within normal limits. Meningeal biopsy showed necrotizing granulomas with Langhans giant cells. The patient was started on antitubercular treatment. He responded well to antitubercular treatment and steroids with near-to-complete neurological recovery except for left sensorineural hearing loss at 10 months. Follow-up MRI at 24 months showed almost complete resolution of meningeal enhancement (Figure 2).

## Discussion

Hypertrophic pachymeningitis is a rare inflammatory disorder that was first described by Charcot and later by Naffziger and Stern [5]. Tuberculosis, a great masquerader of present times, is an important cause of pachymeningitis, especially in developing countries [6]. The peak incidence is seen commonly in the sixth decade. The chronic inflammatory response incites meningeal thickening resulting in compression of anatomic structures such as nerves and vessels at foramina leading to cranial nerve palsies. MRI brain is the modality of choice for the diagnosis of pachymeningitis. The radiological features of pachymeningitis include dural thickening, dural mass, sinus thrombosis, or venous congestion with white matter changes [7]. Continuous dural enhancement on MRI brain is one of the definitive features of pachymeningitis, as seen in our patient. Although the radiological features are characteristic of pachymeningitis, they may not reveal the underlying cause. A step-wise workup including CSF analysis and vasculitis panel can be helpful to recognize the precise etiology. A meningeal biopsy may be essential if the other diagnostic tests are inconclusive of the cause. In our case, a meningeal biopsy was required as there was insufficient evidence of a particular etiology [1]. Tubercular pachymeningitis responds well to an antitubercular regimen with complete clinical recovery in one to two years [8]. Some idiopathic cases of pachymeningitis respond well to antituberculous therapy, suggesting that a proportion of case is due to undiagnosed tuberculosis. Hence, treatment with antituberculous medications and steroids should be considered in cases of idiopathic pachymeningitis [7-10].

## Conclusions

Hypertrophic pachymeningitis is an unusual form of extrapulmonary tuberculosis that should be strongly suspected in patients with chronic headaches and focal neurological deficits, especially if they are from tuberculosis-endemic regions. It is a life-threatening condition that should be diagnosed and treated promptly. MRI findings of dural thickening and CSF analysis may help with the diagnosis. A biopsy of the meninges can help confirm the diagnosis. Tuberculous pachymeningitis responds well to treatment with antitubercular medications and steroids.
